# Melanoma expression of matrix metalloproteinase-23 is associated with blunted tumor immunity and poor responses to immunotherapy

**DOI:** 10.1186/s12967-014-0342-7

**Published:** 2014-12-10

**Authors:** Duane Moogk, Ines Pires da Silva, Michelle W Ma, Erica B Friedman, Eleazar Vega-Saenz de Miera, Farbod Darvishian, Patrick Scanlon, Arianne Perez-Garcia, Anna C Pavlick, Nina Bhardwaj, Paul J Christos, Iman Osman, Michelle Krogsgaard

**Affiliations:** Perlmutter Cancer Center at NYU Langone, New York, NY USA; Department of Pathology, New York University School of Medicine, New York, NY USA; Ronald O. Perelman Department of Dermatology, New York University School of Medicine, New York, NY USA; Interdisciplinary Melanoma Cooperative Group, New York University School of Medicine, New York, NY USA; Instituto Português de Oncologia de Lisboa Francisco Gentil, Lisboa, Portugal; Programme for Advanced Medical Education, Lisbon, Portugal; Department of Surgery, New York University School of Medicine, New York, NY USA; Department of Medicine, New York University School of Medicine, New York, NY USA; Division of Biostatistics and Epidemiology, Weill Cornell Medical College, New York, NY USA

**Keywords:** Matrix metalloproteinase-23, Melanoma, Immunotherapy, Kv1.3, Tumor-infiltrating lymphocytes

## Abstract

**Background:**

Matrix metalloproteinase-23 (MMP-23) can block the voltage-gated potassium channel Kv1.3, whose function is important for sustained Ca^2+^ signaling during T cell activation. MMP-23 may also alter T cell activity and phenotype through cleavage of proteins affecting cytokine and chemokine signaling. We therefore tested the hypothesis that MMP-23 can negatively regulate the anti-tumor T cell response in human melanoma.

**Methods:**

We characterized MMP-23 expression in primary melanoma patients who received adjuvant immunotherapy. We examined the association of MMP-23 with the anti-tumor immune response - as assessed by the prevalence of tumor-infiltrating lymphocytes and Foxp3^+^ regulatory T cells. Further, we examined the association between MMP-23 expression and response to immunotherapy. Considering also an *in trans* mechanism, we examined the association of melanoma MMP-23 and melanoma Kv1.3 expression.

**Results:**

Our data revealed an inverse association between primary melanoma MMP-23 expression and the anti-tumor T cell response, as demonstrated by decreased tumor-infiltrating lymphocytes (TIL) (*P* = 0.05), in particular brisk TILs (*P* = 0.04), and a trend towards an increased proportion of immunosuppressive Foxp3^+^ regulatory T cells (*P* = 0.07). High melanoma MMP-23 expression is also associated with recurrence in patients treated with immune biologics (*P* = 0.037) but not in those treated with vaccines (*P* = 0.64). Further, high melanoma MMP-23 expression is associated with shorter periods of progression-free survival for patients receiving immune biologics (P = 0.025). On the other hand, there is no relationship between melanoma MMP-23 and melanoma Kv1.3 expression (P = 0.27).

**Conclusions:**

Our data support a role for MMP-23 as a potential immunosuppressive target in melanoma, as well as a possible biomarker for informing melanoma immunotherapies.

**Electronic supplementary material:**

The online version of this article (doi:10.1186/s12967-014-0342-7) contains supplementary material, which is available to authorized users.

## Background

Melanoma is a highly immunogenic tumor [1], yet tumor progression nevertheless occurs in immunocompetent patients, which suggests the existence of immune-regulatory mechanisms within the tumor. Tumors can evade immune-mediated destruction through the release of soluble factors that redirect the immune response as well as via mechanisms that limit or inhibit the infiltration or the function of tumor-infiltrating lymphocytes (TILs) [1-3]. Many therapeutic strategies for melanoma have therefore been developed to augment anti-tumor immunity by targeting immunosuppressive mechanisms. Treatments aimed at these mechanisms, such as cytotoxic T lymphocyte-associated antigen-4 (CTLA-4) and programmed cell death 1 (PD-1), work to unrestrain pre-existing TILs from immunosuppressive checkpoints [1]. Select subsets of patients respond favorably to immune-based therapies, but given the morbidity associated with these treatments, clinicopathological criteria are needed to better identify those patients who could benefit and to optimize their immunotherapeutic strategy. Identification of new modulators of immune resistance may also lead to development of anti-melanoma therapeutics that are favorable to patients that are unresponsive to other treatments, or that may act as adjuvants to complement existing therapies to further improve patient outcomes.

Matrix metalloproteinases (MMPs) are a family of zinc- and calcium-dependent proteolytic enzymes that may be either membrane anchored or secreted [4]. The major function of MMPs is degradation of extracellular matrix (ECM) components [5], which can play a role in cancer progression by promoting tumor growth, infiltration and angiogenesis [6]. All MMPs share the common features of an N-terminal signal peptide that directs it to the secretory pathway, a catalytic domain containing a zinc ion in the active site, and a prodomain that interacts with the active site to block enzymatic activity until its removal [4,7]. MMPs also function to cleave non-matrix proteins, including surface receptors, and to activate chemokines and cytokines [4] – mechanisms that have been implicated in a number of other diseases, including arthritis, vascular disease, and Alzheimer’s disease [7,8].

MMP-23 is a membrane-anchored MMP, distinguished from other MMPs in that its N-terminal pro-domain (MMP-23-PD) lacks the enzymatic inhibitory sequence and the characteristic C-terminal hemopexin domain is replaced by an immunoglobulin-like cell adhesion molecule domain [4]. Full-length MMP-23 is found predominantly in perinuclear and endoplasmic reticulum (ER) membranes [9,10], and a single cleavage results in removal of the MMP-23-PD, activation and secretion from the cell [10]. Prior to cleavage, the MMP-23-PD may interact with Kv1.3 potassium channels and regulate their surface expression [11]. Further, MMP-23 also contains a toxin-like domain (MMP-23-TxD) immediately following the catalytic domain [12], which, upon secretion of active MMP-23, may block Kv1.3 channels on proximal cells [11]. MMP-23 therefore has the ability to interact with Kv1.3 in two distinct mechanisms that may affect Kv1.3 membrane expression or function.

The physiological effects of blocking Kv1.3 channels on autoreactive T cells have been demonstrated in the context of autoimmune diseases, including rheumatoid arthritis, type-1 diabetes mellitus, and multiple sclerosis [13,14], where selective blocking may reduce unwanted autoimmune responses. Therefore, MMP-23 has the potential to affect cellular processes through *in cis* Kv1.3 trapping, *in trans* extracellular Kv1.3 blocking [11,12], or through cleavage of proteins affecting cytokine and chemokine signaling [4]. However, it is unclear if MMP-23 expression in melanoma or other cancers can affect disease progression through these mechanisms.

The focus on tumor MMPs in cancers, including melanoma, breast, prostate, lung, and colon cancer, has generally been on their ability to mediate microenvironmental changes to the ECM that regulate cancer progression [15-17]. However, MMPs also play a role in the regulation of anti-tumor immune responses [6]. For example, MMP cleavage of necrosis factor-alpha promotes NF-κβ signaling, resulting in recruitment of immune cells [18]. Similarly, MMPs can influence T cell phenotype - active MMP-2 induces Th2 skewing by blocking IL-12 and inducing OX40L on dendritic cells [19]. While not generally used as a diagnostic marker of cancer, MMP expression has been widely studied as a potential prognostic marker for a number of cancers [7]. Increased tumor expression of MMP-1, MMP-2, MMP-7, or MMP-9 in lung cancers [20-22], and increased expression of MMP-1 and MMP-9 in breast cancer [23,24] is associated with poor patient survival. MMPs, therefore, may be suitable as both biomarkers for cancer prognosis and as therapeutic targets. In melanoma, increased expression of MMP-2 is associated with high invasiveness and melanoma progression [25,26], while expression of MMP-9 was associated with metastasis [27]. However, inhibitors designed for general targeting of MMPs have not seen clinical success, and have lead to a number of undesirable side effects [28], highlighting the need for the development of inhibitors with specific targeting properties.

In this study, we characterize for the first time MMP-23 expression in human melanomas as it relates to anti-tumor immunity and clinical response to immunotherapy. Our data support a role for MMP-23 in blunting the anti-tumor response. Further, our data also support a role for MMP-23 in diminishing melanoma patient responses to immune biologic immunotherapies. This highlights the potential use of MMP-23 melanoma expression as a predictive biomarker for the selection of therapeutic adjuvants and as a potential therapeutic target.

## Methods

### Research design

Primary melanoma tissues obtained before the start of immunotherapy were retrieved from patients enrolled in the Interdisciplinary Melanoma Cooperative Group, a prospectively collected clinicopathologic-biospecimen database at New York University Medical Center [29], between August 2002 and December 2008. Patients were treated with immune-based therapeutics after primary resection or at recurrence. Immunotherapies were categorized as immune biologics (IFN-α, IL-2, GM-CSF) or vaccines (dendritic cell-, peptide-). Informed consent was obtained from all patients at the time of enrollment. Demographic and clinicopathologic information collected included age at pathological diagnosis, gender, primary tumor thickness (mm), ulceration status, mitosis (absent vs. present), histotype, anatomic site, TILs (absent vs. present: non-brisk, brisk as identified by characteristic lymphocytic morphology on hematoxylin-and-eosin staining) [30], recurrence status, and melanoma status at last follow-up (December 2010). TILs were defined as brisk when present throughout the vertical growth phase (i.e. large dermal aggregates of melanoma over 15–25 cells wide) or present and infiltrating across the entire base of the vertical growth phase [30]. All research was approved by the NYU School of Medicine’s Office of Science and Research Institutional Review Board (“Development of an NYU Interdisciplinary Melanoma Cooperative Group: A Clinicopathological Database”, IRB Study # i10362).

### Immunohistochemical analysis

Immunohistochemistry was performed using rabbit polyclonal anti-human MMP-23 - carboxyterminal end (ab39087, Abcam, Cambridge, MA, USA), anti-Kv1.3 (APC-002, Alomone Labs, Ltd., Jerusalem, Israel), and mouse monoclonal anti-human Foxp3 (clone 236A/E7) (eBioscience, San Diego, CA, USA) antibodies on formalin-fixed, paraffin-embedded primary melanoma tissues to detect MMP-23 expression by melanoma cells, Kv1.3 potassium channel expression on tumor cells, and Foxp3^+^ T_regs_, respectively. In brief, after deparaffinization and rehydration, heat-induced epitope retrieval for MMP-23, Kv1.3, and Foxp3 were performed in 0.01 M citrate buffer, pH 6.0, in a 1,200-watt microwave oven at 100% power for 20, 10, and 10 minutes, respectively. Sections were cooled in tap water for 5 minutes, quenched in 0.3% hydrogen peroxide for 30 minutes, washed with PBS, and incubated for 30 minutes with diluted normal blocking serum prepared from goat serum for MMP-23 and horse serum for Foxp3 while a blocking solution containing 5% bovine serum albumin, 0.1% sodium azide, and 5% goat serum was used for Kv1.3. Slides were then incubated with each primary antibody diluted in buffer (MMP-23, 1:100; Kv1.3, 1:50; Foxp3, 1:500) at room temperature for 1 hour and at 4°C overnight, after which they were washed in buffer and incubated with diluted biotinylated secondary antibodies (goat anti-rabbit at 1:500 for both MMP-23 and Kv1.3-stained sections; horse anti-mouse at 1:500 for Foxp3-stained sections, Vector Laboratories, Burlingame, CA, USA) for 1 hour. Avidin-biotinylated horseradish peroxidase complexes diluted at 1:500 (ABC reagent, Vector Laboratories) were added. MMP-23 and Kv1.3 staining were both visualized with peroxidase (ImmPACT™ NovaRED™ Peroxidase Substrate, Vector Laboratories) and diaminobenzidine (DAB substrate kit, Vector Laboratories) was used to visualize Foxp3 staining. Sections were washed in distilled water, counterstained with hematoxylin, dehydrated, and then mounted with permanent media. Appropriate positive and negative controls were included with study sections as well. Specificity of the MMP23 antibody was shown by competition experiment with immunizing peptide (Abcam ab41122) (Additional file 1: Figure S1). MMP23 antibody at a dilution of 1 μg/ml was pre incubated with 2 μg/ml of immunizing peptide for 1 h at room temperature before the application to the tissue, as described above. MMP23 antibody was also assessed by Western blot using 10 μg of protein extracted from melanoma tissues, melanoma cell line, or placenta as positive control, and probed with anti-MMP-23 antibody at 1:5000 dilution (Additional file 2: Figure S2).

**Figure 1:**
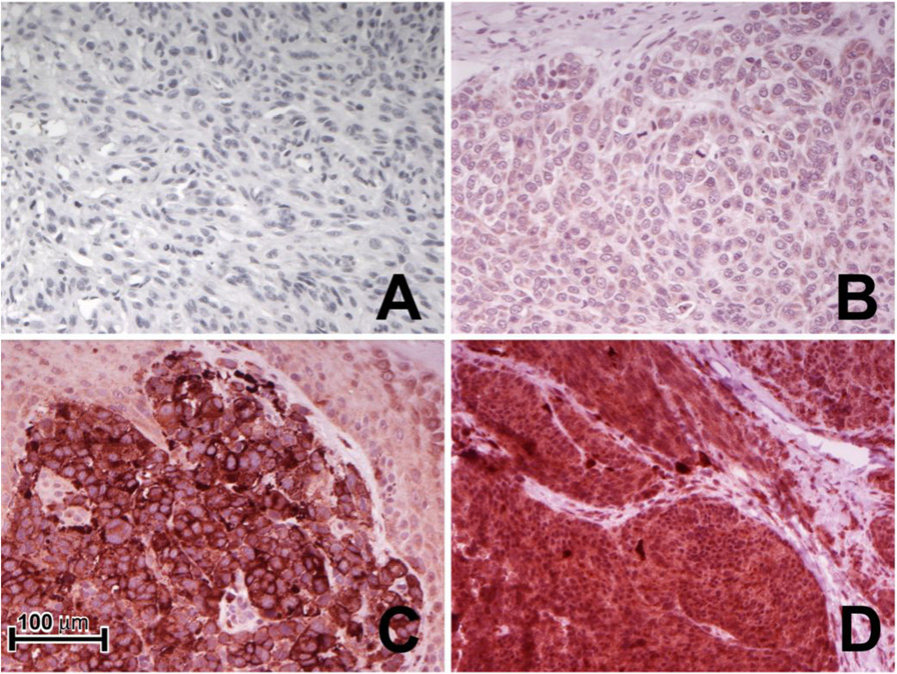
**Representative melanoma MMP-23 immunohistochemical scoring. (A)** Composite score = 0; **(B)** Composite score = 3 (intensity = 1, distribution = 2) with faint cytoplasmic immunopositivity; **(C)** Composite score = 4 (intensity = 2, distribution = 2) with intense cytoplasmic reactivity; **(D)** Composite score = 5 (intensity = 3, distribution = 2) with very intense cytoplasmic reactivity (400X).

An attending pathologist (F.D.) blinded to all clinical data scored the slides for MMP-23, Foxp3, and Kv1.3 expression. Tumor MMP-23 expression was scored for staining intensity (0 = none, 1 = faint, 2 = intense, 3 = very intense) and distribution (0 = none, 1 = focal (<50%), 2 = diffuse (≥50%)), which were summed to generate a composite score for each case as illustrated in Figure 1. Foxp3 expression was scored as the absolute number of positively stained cells with characteristic lymphocytic morphology in a representative high-power field (0.2 mm^2^), and Kv1.3 expression was scored as the absence or presence of Kv1.3 staining on melanoma cells. Each representative high-power field was selected by scanning each slide at 100x to identify the field with the highest antibody expression.

### Statistical analysis

Descriptive statistics were calculated for MMP-23 expression, Kv1.3 expression, and clinicopathologic variables. Univariate associations between MMP-23 expression and continuous clinicopathologic variables were assessed by the ANOVA test or Kruskal-Wallis test, as appropriate. Univariate associations between MMP-23 expression and categorical clinicopathologic variables (including recurrence) were assessed by the chi-square test or Fisher’s exact test, as appropriate. Progression-free survival was defined as the time from immunotherapy to recurrence. Event-time distributions were estimated with the use of the Kaplan–Meier method. Categories of high and low MMP-23 expression were explored in various analyses. All p-values are two-sided with statistical significance evaluated at the 0.05 alpha level. All analyses were performed in SPSS Version 21.0 (SPSS Inc., Chicago, IL).

## Results

### Patient selection and treatment

Table 1 illustrates the demographic and clinicopathological characteristics of the cohort of primary melanoma patients studied. Primary melanoma specimens acquired prior to the initiation of immunotherapy were examined for each of these patients (n = 71). Immunotherapy was given after primary resection (n = 40) or at recurrence (n = 31) (Table 1). Patients were treated with a variety of immunotherapies, categorized as immune biologics (n = 38; IFN-α, IL-2, GM-CSF) or vaccines (n = 33; dendritic cell-, peptide-). Informed consent was obtained from all patients at the time of enrollment. Median follow-up time from the date of pathological diagnosis was 6.3 years (range: 1–10 years). Melanoma was the cause of death for 38/39 patients who died during follow-up.

**Table 1:** **Demographic and clinicopathologic characteristics of melanoma patients treated with immunotherapy**

**Variable**	**Number of patients (n = 71)**
**Age at diagnosis** (years)	
Median (Range)	55 (21–80)
**Gender**	
Male	40 (56.3%)
Female	31 (43.7%)
**AJCC stage at diagnosis**	
I	5 (7.1%)
II	26 (36.6%)
III	40 (56.3%)
**Primary TILs**	
Absent	16 (22.6%)
Present	52 (73.2%)
Non-brisk	29
Brisk	23
Unclassified	3 (4.2%)
**Immunotherapy setting** ^**a**^	
Adjuvant	40 (56.3%)
At Recurrence	31 (43.7%)
**Type of immunotherapy**	
Immune biologic	38 (53.5%)
IFN-α	21
IL-2, IL-18	4
GM-CSF	12
Other	1
Vaccine	33 (46.5%)
Dendritic cell	11
Peptide	22

*Abbreviations: AJCC* American joint committee on cancer, *TILs* Tumor-infiltrating lymphocytes, *NOS* Not otherwise specified.

^a^Patients who received immunotherapy in both settings (n=6).

### Melanoma MMP-23 expression does not correlate with melanoma Kv1.3 expression

Tumor-derived MMPs in melanoma and other cancers can mediate microenvironmental changes regulating cancer progression [15-17]. Melanoma cells can express Kv1.3 [31], although the role of tumor Kv1.3 expression is unclear. However, in prostate cancer, reduced tumor cell Kv1.3 expression is associated with poor clinical outcome [32]. We therefore hypothesized that melanoma Kv1.3 surface expression may be inhibited by *in cis* MMP-23 trapping of Kv1.3 at the ER [12]. To examine the possible effect of melanoma MMP-23 expression on tumor Kv1.3 expression, 20 primary melanomas were evaluated for both MMP-23, as measured by a composite score of MMP-23 staining intensity and distribution (Figure 1), and Kv1.3 expression. Kv1.3 expression was absent in 10/15 (67%) primary tumors that had high MMP-23 expression as well as in 5/5 (100%) primary melanomas that had absent or low MMP-23 expression (*P* = 0.27) (Table 2). Our data therefore suggest that tumor MMP-23 expression does not directly affect tumor Kv1.3 expression as would occur via an *in* cis mechanism. Our data did not demonstrate an association between tumor Kv1.3 expression and clinical outcome as assessed by recurrence following immunotherapy. Recurrence was observed in 11/15 (73%) patients whose primary tumors were absent Kv1.3 expression, and similarly in 4/5 (80%) patients whose primary melanomas stained positive for Kv1.3 (*P* = 0.99) (Table 2). Therefore, there does not appear to be a link between melanoma MMP-23 and Kv1.3 expression, and further, melanoma Kv1.3 expression does not correlate with immunotherapy outcome.

**Table 2:** **MMP-23 expression and recurrence according to Kv1.3 expression.**

	**MMP-23 composite score** ^**a**^			**Recurrence** ^**b**^		
**Kv1.3 expression**	0-2	3-4		Yes	No	
Yes	0 (0%)	5 (33%)	5 (100%)	4 (27%)	1 (20%)	5 (100%)
No	5 (100%)	10 (67%)	15 (100%)	11 (73%)	4 (80%)	15 (100%)
**Total**	5 (100%)	15 (100%)		15 (100%)	5 (100%)	

^a^P = 0.27; ^b^P = 0.99.

### Higher melanoma MMP-23 expression is associated with a blunted anti-tumor immune response

In the absence of *in cis* regulation of melanoma Kv1.3, MMP-23 also has the potential to act *in trans* through cleavage of surface protein or blocking Kv1.3 channels on nearby cells, including TILs. Blocking of Kv1.3 on T cells can diminish the driving force for calcium influx, thereby reducing activation-induced proliferation and motility [33]. Furthermore, prolonged inhibition of Kv1.3 can prevent T cells from receiving activation or survival signals, resulting in death due to cytokine deprivation [14]. Cleavage of non-ECM proteins may affect cytokine and chemokine signaling, and has the potential to skew T cell phenotype [18,19]. We therefore considered that melanoma MMP-23 expression could have a role in regulating anti-tumor immune responses.

**Figure 2:**
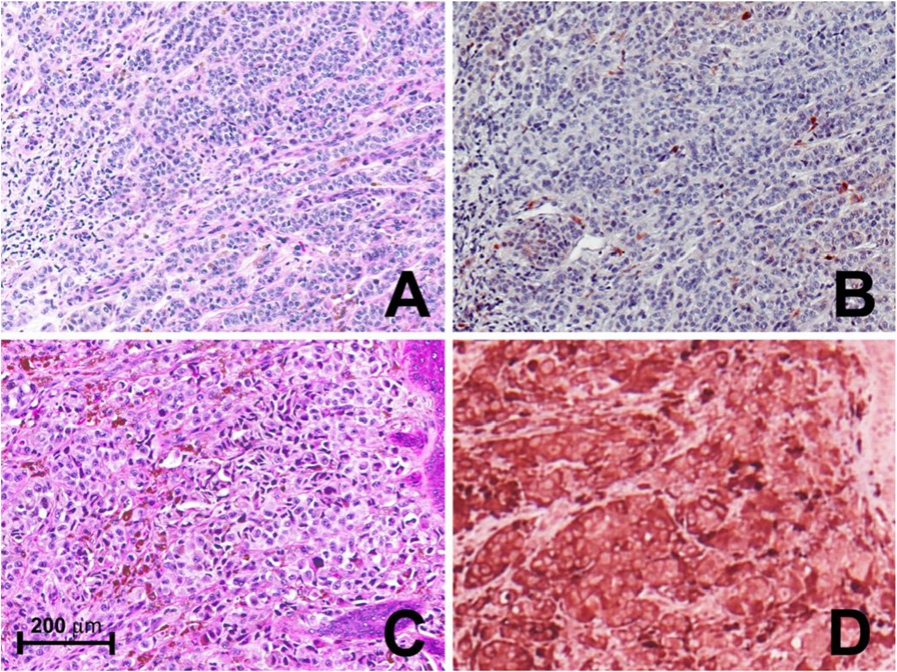
**Intensity of lymphocytic infiltration decreases with increased melanoma MMP-23 expression.** Hematoxylin-and-eosin-stained primary melanoma specimens showing a representative brisk **(A)** and absent **(C)** lymphocytic infiltrate and the corresponding consecutive MMP-23-stained sections (**B** and **D**, respectively) with MMP-23 composite scores of 2 and 4, respectively (80X).

To evaluate the relationship between primary melanoma MMP-23 expression and anti-tumor immunity, we compared melanoma MMP-23 expression with two separate measures of the strength of the intrinsic anti-tumor immune response – the presence and the degree of TIL infiltration [30] – at the time of primary resection (Figure 2). We observed that increased melanoma MMP-23 expression is inversely associated with the presence of TILs (presence = 79.4% for melanomas with low MMP-23 expression and 53.8% for melanomas with high MMP-23 expression, *P* = 0.05). Quantification of the intensity of lymphocytic infiltration also showed a significant inverse correlation between MMP-23 expression and a brisk lymphocytic response (brisk TILs = 65.0% for melanomas with low MMP-23 expression and 25.0% for melanomas with high MMP-23 expression, *P* = 0.04). These results suggest a role for tumor-derived MMP-23 in the suppression of anti-tumor immune responses.

**Figure 3:**
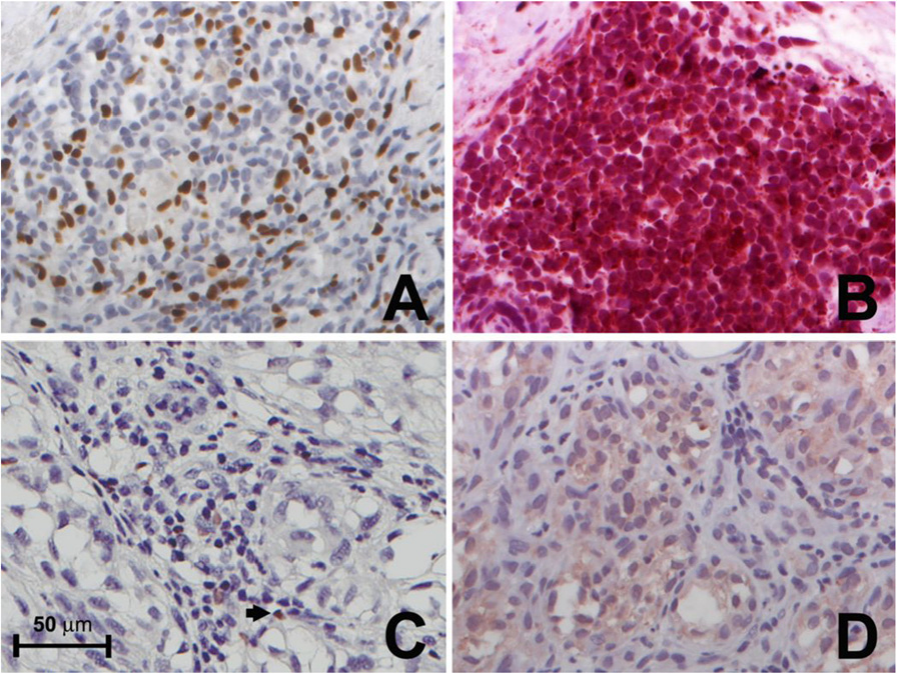
**Foxp3** ^**+**^ **T**_**regs**_ **in the primary tumor increases with higher melanoma MMP-23 expression. (A)** Heterogeneous nuclear positivity for Foxp3 in TILs from a primary melanoma with a high MMP-23 composite score and 100 Foxp3^+^ T_regs_/high-power field. **(C)** Nuclear reactivity for Foxp3 limited to a few TILs (5 Foxp3^+^ T_regs_/high-power field) (arrow) from a primary melanoma with no MMP-23 expression (200X). **(B, D)** Corresponding consecutive MMP-23-stained sections of **A** and **C**, respectively.

In addition to tumor-specific T-lymphocytes, the TIL population is also comprised of immunosuppressive Foxp3^+^ regulatory T cells (T_regs_) that play an important role in immune evasion [34,35]. The accumulation of T_regs_ in the tumor microenvironment can be attributed to a number of factors, including the local expression and secretion of factors affecting T_reg_ migration and retention, the expansion of naturally occurring T_regs_, or the *de novo* generation of induced T_regs_ [36]. To investigate the potential role of melanoma MMP-23 in contributing to conditions favorable to T_regs_, we assessed the relationship between MMP-23 expression and T_reg_ prevalence, as determined by the number of Foxp3^+^ cells (Figure 3). We observed a trend towards an increased number of T_regs_ in primary melanomas with higher MMP-23 expression (53.1 ± 33.8 in melanomas with high MMP-23 expression and 35.0 ± 25.1 in melanomas with low MMP-23, *P* = 0.07). This suggests a potential role for MMP-23 in skewing TIL phenotype. Combined, these results suggest that MMP-23 plays a role in blunting the immune response to melanoma as it affects the prevalence, distribution, and composition of TILs in favor of tumor immune evasion.

### Higher MMP-23 expression is associated with resistance to immune biologic immunotherapy

To further evaluate the potential role of MMP-23 in tumor immune escape, we examined the relationship between primary melanoma MMP-23 expression and clinical response to immunotherapy, as measured by the rate of recurrence. Considering the entire cohort of patients – receiving immunotherapy after primary resection or at recurrence – subsequent recurrence was detected in 11/19 (59%) patients with low melanoma MMP-23 expression (composite score = 0–2) compared with 42/52 (81%) patients high melanoma MMP-23 expression (composite score = 3–4) (*P* = 0.067) (Table 3). This trend holds when considering only patients who had received immunotherapy after primary resection (vaccines (n = 21), immune biologics (n = 19)), where the recurrence rate was lower in patients with low melanoma MMP-23 expression (5/11; 46%) compared with patients with high melanoma MMP-23 expression (23/29; 79%) (*P* = 0.056) (Table 3). These trends suggest that MMP-23 may play a role in regulating immune responses in the context of immunotherapy.

**Table 3:** **Analysis of melanoma recurrence according to MMP-23 expression in the entire cohort and the subset of patients who received immunotherapy after primary resection**

	**MMP-23 composite score**			
	**Entire cohort** ^**a**^		**Immunotherapy after primary resection** ^**b**^	
	**0 - 2**	**3 - 4**	**0 - 2**	**3 - 4**
**Recurrence**				
Yes	11 (59%)	42 (81%)	5 (46%)	23 (79%)
No	8 (41%)	10 (19%)	6 (54%)	6 (21%)
**Total**	19 (100%)	52 (100%)	11 (100%)	29 (100%)

^a^P = 0.067; ^b^P = 0.056.

Different classes of immunotherapies target different components of the anti-tumor immune response. Vaccines target CD8^+^CCR7^+^ central memory T cells (T_CM_) and immune biologics target CD8^+^CCR7^−^ effector memory T cells (T_EM_) [37-39]. Therefore, we evaluated the clinical outcomes of primary melanoma patients treated with vaccines and immune biologics separately to determine if the observed relationship between tumor MMP-23 expression and recurrence could be attributed to an effect on a specific T cell subset (Table 4). Considering all patients who received vaccine therapy (after primary resection or at recurrence), no significant difference in recurrence was detected between patients with low melanoma MMP-23 expression and those with high melanoma MMP-23 expression. This is also true when considering all patients who received immune biologics (after primary resection or at recurrence). However, when we focused our analysis on patients treated with immune biologics only after primary resection, and therefore as primary adjuvant therapy, higher primary melanoma MMP-23 expression was associated with increased recurrence, as recurrence was detected in 1/4 (25%) patients with low melanoma MMP-23 expression, compared with 13/15 (87%) patients with high melanoma MMP-23 expression (*P = 0.037*). In contrast, the level of tumor expression of MMP-23 in patients treated with vaccine immunotherapies at primary resection was not associated with recurrence (*P = 0.64*).

**Table 4:** **Stratified analysis of melanoma recurrence risk by type of immunotherapy (immune biologics and vaccines) in the patients who had primary adjuvant immunotherapy**

	**MMP-23 composite score**			
	**Immune biologics** ^**a**^		**Vaccines** ^**b**^	
	**0 - 2**	**3 - 4**	**0 - 2**	**3 - 4**
**Recurrence**				
Yes	1 (25%)	13 (87%)	4 (57%)	10 (71%)
No	3 (75%)	2 (23%)	3 (43%)	4 (29%)
**Total**	4 (100%)	15 (100%)	7 (100%)	14 (100%)

^a^ *P* = 0.037; ^b^ *P* = 0.64.

**Figure 4:**
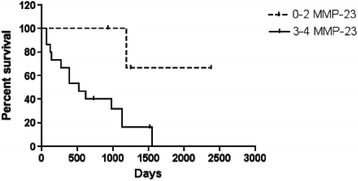
**Progression-free survival of melanoma patients receiving immune biologics.** Patients treated with immune biologics were monitored for recurrence from the time treatment, and grouped based on expression of MMP-23 prior to initiation of treatment (dashed line – MMP-23 composite score = 0–2; solid line – MMP-23 composite score = 3–4). P = 0.025.

To further investigate the affect of MMP-23 on patients receiving immune biologics, we next compared the progression free survival between patients with high versus low melanoma MMP-23 expression (Figure 4). These data revealed that high melanoma MMP-23 expression is associated with shorter periods of progression-free survival (*P = 0.025*). Together, these results suggest that, in patients treated with immune biologics, high MMP-23 expression levels augment the anti-tumor immune response to both increase the likelihood of recurrence and shorten the time to recurrence. Although statistically significant, we acknowledge that larger sample sizes are required to test these results with greater statistical rigor. These results suggest that high tumor expression of MMP-23 confers a level of resistance to immune biological therapy when given as a primary adjuvant. Interestingly, tumor MMP-23 seems to affect specifically T_EM_ cells, as immune biologics including IFN-α, IL-2, and GM-CSF function by targeting T_EM_ expansion.

## Discussion

We examined the expression of MMP-23 in melanoma and our data suggest that MMP-23 represents an immune escape mechanism and potential immunotherapeutic target. Our data show that melanoma MMP-23 expression correlates with a diminished anti-tumor T cell response and higher numbers of T_regs_ in the TIL population. These data together support a role for tumor MMP-23 in modulating both the infiltration and activation of tumor-reactive lymphocytes.

Anti-tumor T cells comprise the majority of the tumor lymphocytic infiltrate, and our study shows that the number, intensity of infiltration, and composition of TILs are negatively affected by melanoma MMP-23 expression. Increased tumor expression of MMP-23, therefore, mediates intrinsic tolerance with the potential to confer resistance against immunotherapy. As such, an evaluation of melanoma MMP-23 expression may be particularly useful in selecting candidates for adoptive T cell therapy, the success of which depends on the harvest, expansion and immunophenotype of TILs [40]. Furthermore, our results suggest the clinical relevance of assessing primary melanoma MMP-23 expression prior to initiating adjuvant treatment with immune biologics, as melanoma MMP-23 expression negatively correlates with response to adjuvant immune biologic therapy. Immune biologics, such as IFN-α, IL-2, and GM-CSF, preferentially expand T_EM_ cells, whereas vaccines induce T_CM_ responses. The dependence of immune biologics but not vaccines on MMP-23 expression, suggests that MMP-23 specifically affects anti-tumor T_EM_ responses. When activated, T_EM_ express an increased number of Kv1.3 channels [37,38] and sustained and complete activation is dependent of their function. On the other hand, T_CM_ are characterized by low Kv1.3 expression and are not dependent on Kv1.3 function for activation [14]. Therefore, MMP-23 may inhibit anti-tumor T_EM_ responses in adjuvant immune biologic therapies through *in trans* blocking of T_EM_ Kv1.3 channels. This could explain the lack of an observed association between melanoma MMP-23 expression and recurrence among patients treated with adjuvant vaccine therapy. Patient stratification based on MMP-23 expression, type of adjuvant therapy, and timing of treatment (i.e. at the time of primary resection) significantly reduced the number of patients in specific groups. The statistical analysis of these data, specifically patients with low MMP-23 expression receiving immune biologic therapy at primary resection (n = 4), is presented with the acknowledgement that the conclusions drawn from these data would benefit from larger sample sizes.

The observed trend toward an increased number of T_regs_ within tumors that expressed high levels of MMP-23 suggests that this may contribute further to suppression of anti-tumor T cell responses. A number of studies have reported a correlation between T_reg_ infiltration and patient prognosis, where high percentages of T_regs_ in both primary melanomas and lymph node metastases correlated with increased recurrence and decreased survival rates [41-43], although this correlation was not observed in other studies [44,45]. T_reg_ accumulation in the tumor may be driven by a number of factors, including local chemokine and integrin-ligand expression [46], and immunosuppressive factors promoting expansion of existing T_regs_ or generation of induced T_regs_ [47]. How increased MMP-23 expression might function to increase the tumor T_reg_ population is unclear. Recent work by Godefroy and colleagues [19] showed that MMP-2 in melanoma degrades type I IFN receptor, effectively preventing IL-12p35 production, and skews CD4 T cells towards a Th2 phenotype. It is possible that MMP-23 activity alters signaling pathways controlling T_reg_ expansion or de novo production of induced T_regs_. A Kv1.3 knock-out induced EAE mouse model of infection showed that CD4+ T cells expressed greater levels of IL-10 and lower levels of IL-17 and IFN-γ, and suppressed proliferation of wild-type CD4+ T cells, suggesting a skewing toward a regulatory phenotype [48], although an increase in the number of Foxp3+ cells was not observed.

While our data suggest a role for melanoma MMP-23 in blunting anti-tumor immunity by selectively blocking T cell Kv1.3 channels, we also explored the possibility that melanoma MMP-23 may act *in cis* by trapping Kv1.3 in the ER and preventing surface expression. Of the primary melanomas evaluated for Kv1.3 surface expression, most were negative, which may reflect the suppression of Kv1.3 surface expression due to *in cis* MMP-23 trapping [12] or an absence of Kv1.3 expression altogether. However, for our limited sample size, we did not observe a relationship between MMP-23 expression and tumor Kv1.3 surface staining. Kv1.3 channels on melanoma cells, however, have previously been shown to be in close proximity to β1-integrins, such that blockade of Kv1.3 channels dysregulates integrin function and results in loss of cell adherence [31]. Disruption of cell-cell/cell-matrix adhesion is one of many steps in metastatic progression, and evidence from prostate cancer support the association between reduced tumor cell Kv1.3 expression and poor clinical outcome [32]. Studies in breast and colon cancer, in contrast, suggest that blockade of tumor cell Kv1.3 expression is protective [49,50] as it prevents progression through the G_1_/S checkpoint, which requires transient hyperpolarization [51]. In trapping Kv1.3 in the ER, melanoma MMP-23 would likewise alter the tumor cell membrane potential and facilitate the transition to the M phase. Further studies are warranted to explore the possible role of MMP-23 in tumor Kv1.3 suppression.

Inhibition of MMP-23 in combination with other immunotherapies may further augment anti-tumor immunity. With the clinical success of monoclonal antibodies against inhibitory immune checkpoint proteins in melanoma, including CTLA-4 [52] and PD-1 [53], anti-MMP-23 therapy also holds promise as a potential treatment strategy. Yet, such strategies must consider the possibility of off-target effects; thus the normal tissue distribution and physiologic role of MMP-23 must first be understood. Unlike other MMPs, MMP-23 is widely expressed under physiologic conditions, in particular at high levels in the ovary, testis, prostate, and heart and at low levels in the lung, pancreas, and colon [10,54]. Intralesional delivery of MMP-23 inhibitors may therefore be preferred over systemic injection to minimize possible off-target effects. Furthermore, locoregional MMP-23 inhibition has the potential to alter both the number and the composition of the TILs, such that adoptive T cell therapy candidates could benefit from pre-treatment with intralesional MMP-23 inhibitors.

In identifying MMP-23 as a novel immunotherapeutic target, it is important to recognize that inhibition of MMP-23 may result in immune-related adverse events, much like the inhibition of CTLA-4 [55]. Ongoing efforts to identify biomarkers to predict the response to anti-CTLA-4 therapy are under way just as additional studies to identify those patients most likely to benefit from MMP-23 inhibition are needed in parallel to those of the role of MMP-23 in melanoma. Our study provides the foundation for both as data suggest that an assessment of melanoma MMP-23 expression by routine immunohistochemistry may prove useful, much like using immunohistochemistry to determine the estrogen/progesterone receptor status in breast cancer [56]. Yet, the clinical value of assessing melanoma MMP-23 expression in conjunction with Kv1.3 expression remains to be determined.

## Conclusions

Our study suggests that MMP-23 may play a role in tumor-induced immune escape and that melanoma MMP-23 expression represents a novel biomarker and possible immunotherapeutic target. MMP-23 inhibition in combination with other therapeutic agents may be more effective than as monotherapy, yet studies are still needed to elucidate the exact mechanism by which melanomas upregulate MMP-23 to allow the development of a specific MMP-23 inhibitor with a favorable risk-benefit profile.

## Additional files

Additional file 1: Figure S1. Demonstration of MMP-23 antibody specificity by competition with immunizing peptide. Immunostaining by MMP-23 primary antibody plus secondary antibody **(A)**, preincubated with immunizing peptide before addition of primary and secondary antibodies **(B)**, and with secondary antibody only in the absence of primary MMP-23 antibody **(C)**. Additional file 2: Figure S2. Western blot demonstrating detection of MMP23 expression using MMP-23 antibody. **(A)** MMP-23 Western blots using different MMP-23-specific antibodies to probe 10 μg of placenta protein per lane: Lane A) - ab39087, immunogen C terminus of MMP-23, diluted 1:5000; Lane B) ab74215 immunogen peptide derived from the C terminus of human MMP-23 protein, diluted 1:1000; Lane C) ab39086, immunogen peptide corresponding to the hinge region of human MMP-23, diluted 1:5000; and Lane D) ab 53148, immunogen Synthetic peptide derived from human MMP-23, diluted 1:1000. **(B)** 10 μg of protein was extracted from melanoma tissues (Lanes A-D), melanoma cell line (Lane E) or placenta (Lane F) and assessed by Western blot using ant-MMP-23 antibody, ab39087.

## Abbreviations

MMP-23: Matrix metalloproteinase-23
MMPs: Matrix metalloproteinases
MMP-23-PD: MMP-23 pro-domain
MMP-23-TxD: MMP-23 toxin-like domain
TIL: Tumor infiltrating lymphocytes
CTLA-4: Cytotoxic T lymphocyte-associated antigen-4
PD-1: programmed cell death-1
ECM: Extracellular matrix
ER: Endoplasmic reticulum
T_CM_: Central memory T cell
T_EM_: Effector memory T cell
