# Supplementary figures and images for: Melanoma expression of matrix metalloproteinase-23 is associated with blunted tumor immunity and poor responses to immunotherapy
Source: J Transl Med. 2014 Dec 10;12:342. doi: 10.1186/s12967-014-0342-7 (PMC4272770; doi:10.1186/s12967-014-0342-7)

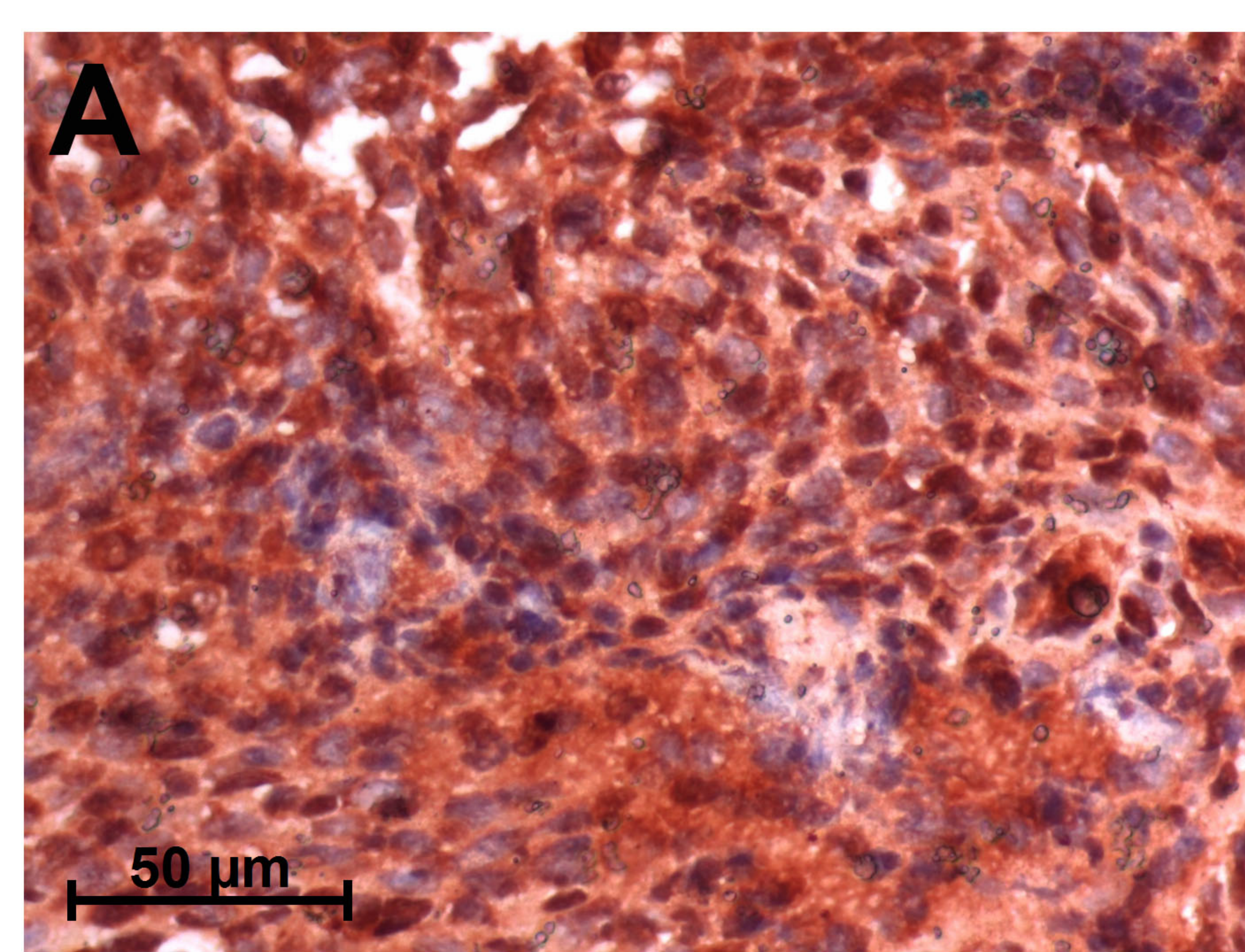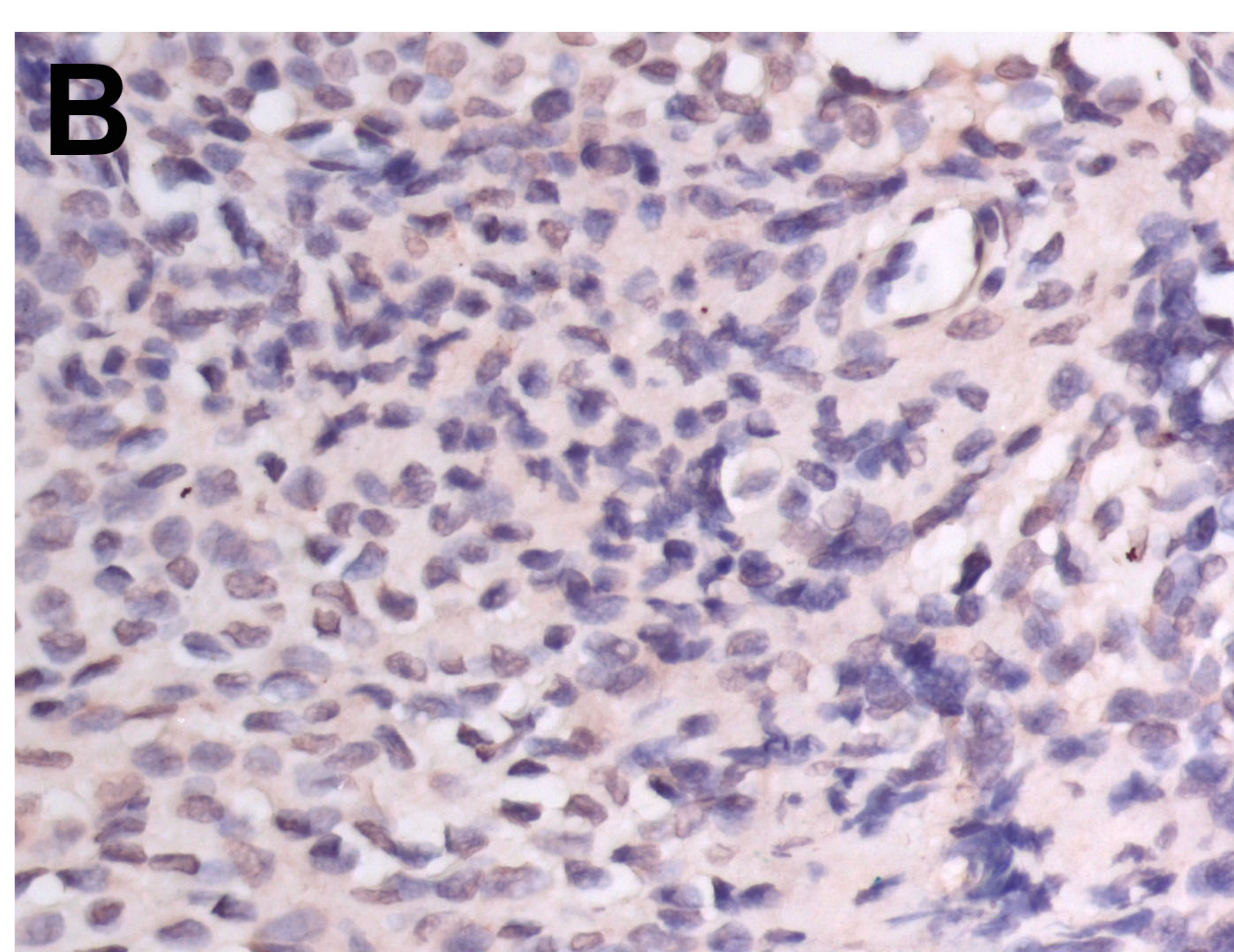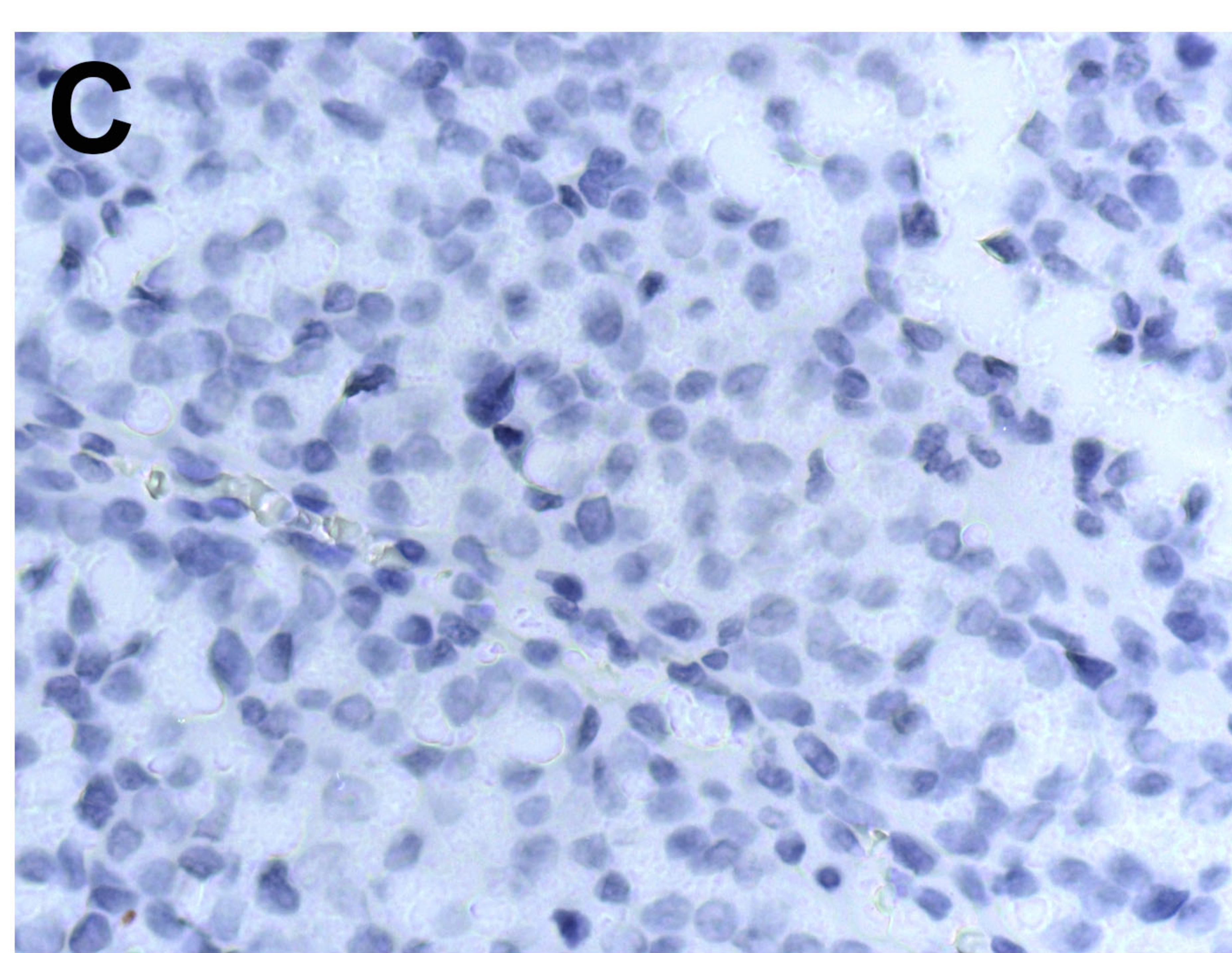

Supplement: Additional file 1: Figure S1. — Demonstration of MMP-23 antibody specificity by competition with immunizing peptide. Immunostaining by MMP-23 primary antibody plus secondary antibody (A), preincubated with immunizing peptide before addition of primary and secondary antibodies (B), and with secondary antibody only in the absence of primary MMP-23 antibody (C). [file 12967_2014_342_MOESM1_ESM.pdf]

**A**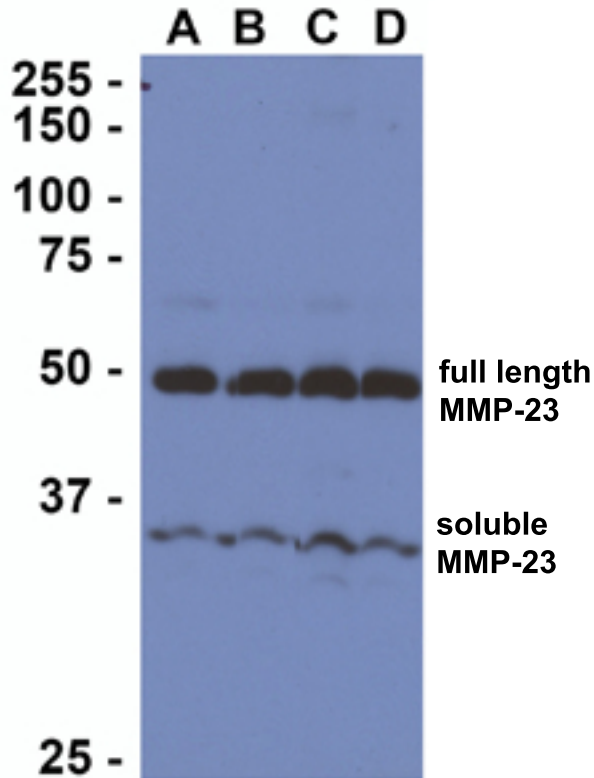**B**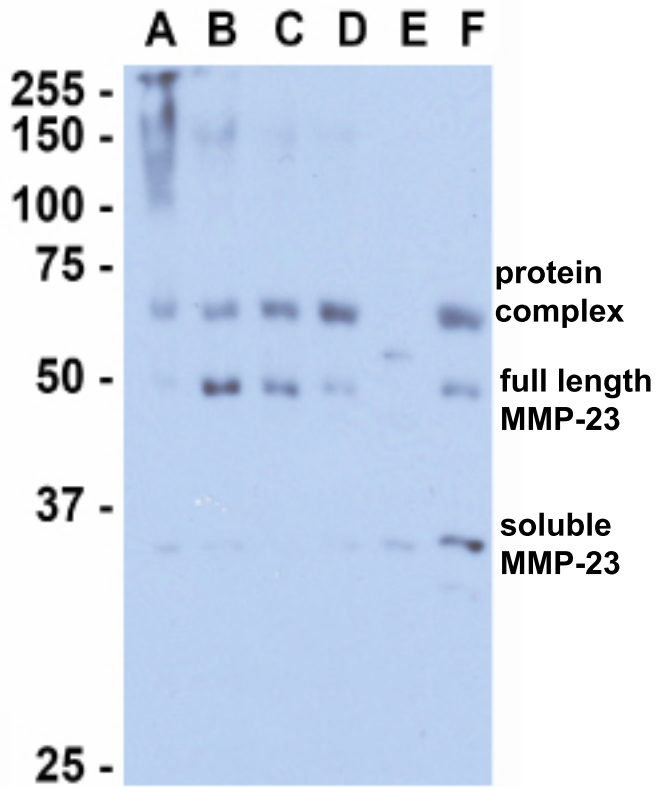

Supplement: Additional file 2: Figure S2. — Western blot demonstrating detection of MMP23 expression using MMP-23 antibody. (A) MMP-23 Western blots using different MMP-23-specific antibodies to probe 10 μg of placenta protein per lane: Lane A) - ab39087, immunogen C terminus of MMP-23, diluted 1:5000; Lane B) ab74215 immunogen peptide derived from the C terminus of human MMP-23 protein, diluted 1:1000; Lane C) ab39086, immunogen peptide corresponding to the hinge region of human MMP-23, diluted 1:5000; and Lane D) ab 53148, immunogen Synthetic peptide derived from human MMP-23, diluted 1:1000. (B) 10 μg of protein was extracted from melanoma tissues (Lanes A-D), melanoma cell line (Lane E) or placenta (Lane F) and assessed by Western blot using ant-MMP-23 antibody, ab39087. [file 12967_2014_342_MOESM2_ESM.pdf]
